# Genetics and Clinical Findings Associated with Early-Onset Myopia and Retinal Detachment in Saudi Arabia

**DOI:** 10.3390/genes16070848

**Published:** 2025-07-21

**Authors:** Mariam M. AlEissa, Abrar A. Alhawsawi, Doaa Milibari, Patrik Schatz, Hani B. AlBalawi, Naif M. Alali, Khaled K. Abu-Amero, Syed Hameed, Moustafa S. Magliyah

**Affiliations:** 1 Research Department, King Khaled Eye Specialist Hospital, Riyadh 11462, Saudi Arabia; mariam_al_eissa@hotmail.com (M.M.A.); kamero@kkesh.med.sa (K.K.A.-A.); syhameed@kkesh.med.sa (S.H.); 2 College of Medicine, Alfaisal University, Riyadh 11533, Saudi Arabia; 3 Public Health Laboratory, Public Health Authority, Riyadh 12382, Saudi Arabia; 4 The Computational Sciences Department at the Centre for Genomic Medicine (CGM), King Faisal Specialist Hospital & Research Centre, Riyadh 11211, Saudi Arabia; 5 Division of Ophthalmology, Department of Surgery, College of Medicine, University of Jeddah, Jeddah 23218, Saudi Arabia; aaalhawsawi@uj.edu.sa; 6 Ophthalmology Department, King Abdullah Medical City, Makkah 24211, Saudi Arabia; doaamilibari@gmail.com; 7 Department of Ophthalmology, Clinical Sciences, Skane University Hospital, Lund University, 221 85 Lund, Sweden; patrik.schatz@med.lu.se; 8 Division of Ophthalmology, Department of Surgery, Faculty of Medicine, University of Tabuk, Tabuk 47311, Saudi Arabia; hb.albalawi@ut.edu.sa (H.B.A.); nmalali@ut.edu.sa (N.M.A.); 9 Vitreoretinal Division, King Khaled Eye Specialist Hospital, Riyadh 11462, Saudi Arabia

**Keywords:** eye, Saudi Arabia, early-onset myopia, retinal detachment

## Abstract

Autosomal recessive types of both syndromic and non-syndromic inherited myopia are common in Saudi Arabia (SA) because many people marry their relatives. The prevalence of syndromic myopathies in SA, like Stickler syndrome (SS), Knobloch syndrome (KS), and Marfan syndrome (MFS), further complicates the disease spectrum. The causative genes linked to the Knobloch, Marfan, and Pierson syndromes are *COL18A1*, *FBN1*, and *LAMB2*, respectively. Additionally, we found recessive types of non-syndromic high myopia that have a high chance of causing retinal detachment, like those linked to *LRPAP1* and *LEPREL1*. In these cases, regular evaluation and early intervention, including prophylactic laser photocoagulation and pars plana vitrectomy, may improve the outcome. Advancements in genetic testing for diagnosis and prevention accelerate detection, facilitate early interventions, and provide genetic counseling. The utilization of artificial intelligence (AI), machine learning (ML), and the advancement of gene therapy offer promising avenues for personalized care. We place a high value on using genetic knowledge to create a national screening program and patient registry aimed at understanding the national burden of myopia, knowing that we have a high rate of consanguinity, which reflects pathogenic homozygous alleles and founder mutations. This initiative will incorporate genetic counseling and leverage innovative technologies, which are crucial for disease management, early identification, and prevention in Saudi Arabia’s healthcare system.

## 1. Introduction

Cultural and socioeconomic status in Saudi Arabia (SA) influenced the genetic landscape, influenced by the high prevalence of consanguineous marriages [1]. The prevalence of genetic diseases in SA is considered highly influenced by a homogeneous genetic pool, leading to a high frequency of autosomal recessive (AR) pathogenic alleles [2,3]. In Saudi Arabia, a significant public health concern is the rise in genetic disease predisposition [4]. This trend might be related to the high rates of consanguineous marriages, which, in turn, increase the risks of high myopia (HM) and childhood rhegmatogenous retinal detachment (RRD) [5,6,7]. The elevated prevalence of AR rare diseases in Saudi Arabia is a result of the significant increase in consanguinity, which increases the probability of pathogenic homozygosity variation, although mutations in genes *LEPREL1*, *LRPAP1*, *LAMB2*, and *COL18A1* were found among unrelated individuals, indicating a population-specific founder effect [8,9]. The distinctive population genetics landscape demands a local database that assesses genetics interpretations which reflect on a tailored screening strategy [1].

Government-led screening programs for newborns exist in SA, but they currently do not screen for high myopia or RRD [6]. The value of early detection is crucial for better outcomes, knowing that the success rate for RRD surgery is 78.3% after a single surgery, with a final rate of anatomic success of 91.5% [7]. Familial screening for rare conditions with an AR pattern of inheritance, especially for patients with a known family history, encourages the implementation of preventive measures to prevent the disease onset in the family and facilitates interventions via new innovative biotechnology like gene therapy [10]. This review outlines the genetic findings detected in Saudi patients and the associated pathological mechanisms of EOM and RRD and integrates data from those studies.

## 2. Disease Overview

### 2.1. Stickler Syndrome

Stickler syndrome (SS, arthro-ophthalmopathy) was first described by Gunnar Stickler in 1965 as a hereditary arthro-ophthalmopathy disease [11]. It is a genetic condition that affects multiple systems in the body and is caused by changes in genes that make collagen, usually passed down from parents to children in an autosomal dominant way, but sometimes in an AR way, and there have also been some cases that occur randomly. Collagen is a key part of the eye’s structure, especially in the sclera and vitreous, which may lead to myopia because of the eye growing longer and changes in the vitreous gel. Collagen is a fundamental protein found in the connective tissues throughout the body, including the various parts of the eye [12]. In the cornea, the precise organization of collagen fibrils, primarily types I and V, is essential for transparency and refractive function [13,14]. Similarly, collagen in the lens (I, III, and IV) contributes to the structural integrity of the lens capsule, ensuring its transparency and flexibility for proper focusing [13,14]. The vitreous body primarily contains type II collagen along with other types of collagens, IX and XI, which help maintain the gel-like consistency necessary for light transmission and retinal support [13,14]. In contrast, the sclera relies mainly on type I and III collagen for its strength and rigidity, ensuring the eye maintains its shape and structural integrity [13,14]. The different types of collagens present in these ocular structures work together to provide both mechanical support and an optimal optical environment necessary for clear vision.

The disease is caused by heterozygous pathogenic variants in *COL2A1*, *COL11A1*, or *COL11A2* or biallelic pathogenic variants in *COL9A1*, *COL9A2*, or *COL9A3*. *COL2A1*, *COL11A1*, or *COL11A2* mutations are associated with autosomal dominant inheritance, whereas mutations in *COL9A1*, *COL9A2*, or *COL9A3* are an AR disease. It is characterized by ocular, craniofacial, auditory, and articular abnormalities in a variable degree [11,15]. Most individuals with pathogenic *COL2A1* variants will present with systemic manifestations of SS; however, those with pathogenic variants in exon 2 may have an ocular-only phenotype due to alternative splicing [16]. Variants in *COL2A1* outside of exon 2 can also cause ocular-only phenotypes [17]. Thus, a diagnosis of SS should be considered in an individual with suggestive ocular findings even with no systemic features [13,16,17].Congenital nonprogressive myopia with or without astigmatism, vitreoretinal degeneration (vitreous syneresis can be either membranous, which is more common in type I, or have a beaded configuration, which is more common in type II, and perivascular lattice degeneration), and RRD are among the most common ophthalmological features [15,18,19,20]. However, between vitreous degeneration morphologies (membranous in type I, beaded in type II) and gene COL subtypes, there is no significant association between the genotype and phenotype. It has been reported by Boysen et al. (2020) [21] that there is no significant difference in RD risk between SS types I and II.

Congenital cataract may also be present, which is typically quadratic lamellar cortical opacity that may not involve the visual axis, and in some individuals, there are developmental anomalies of the anterior chamber angle that increase the risk of glaucoma [15,18,19,20].

Systemic manifestations are widespread and include midface hypoplasia, micrognathia, Pierre Robin syndrome, cleft palate, neurosensory or conductive hearing loss, precocious arthritis, scoliosis, kyphosis, platyspondyly, and joint hypermobility [15,18,19,20]. These ocular and systemic features may overlap and result in variable clinical phenotypes of SS subtypes or sometimes share the phenotypes of other ocular conditions, such as familial exudative vitreoretinopathy (FEVR) [22] or mimic other myopia-related syndromes, such as those caused by recessive mutations in *LEPREL1* [23]. SS is considered the most common cause of inherited RRD in childhood [15,18]. RRD is a significant cause of visual loss in SS patients, typically developing from a giant retinal tear at the base of the pars plana. The risk of RD in SS globally is well known. A questionnaire of cohorts from the UK and US reported an overall risk of RD up to 60% in patients with a clinical diagnosis of one of the SS [20]. This risk reaches up to 70% in patients with genetically proven type 1 SS and decreases to 40% in patients with genetically proven type II [23,24]. However, the literature has not identified any genotype–phenotype correlation to explain the difference in RD risk between SS subtypes. We think this difference might be due to changes in vitreous types, since the membranous type is more often linked to type I SS, while the beading type is more common in type II. Nevertheless, no direct correlation has been established between gene mutations and vitreous phenotype [25]. Another possible explanation is that the risk of RD in individuals with type II SS has been less extensively studied compared to those with type I SS [20]. On the other hand, Boysen et al. [21], in their systematic review and meta-analysis of 37 studies, which included a total of 2324 individuals with SS, reported an overall RD risk of 45%. They found no statistically significant difference in RD prevalence between type I and type II SS (49% vs. 38%), with a *p*-value of 0.31 [26]. The average age of RD in SS was found to be around 30 years [19], and the first detachment usually occurred earlier in Type I SS compared to Type II SS [26].

### 2.2. Stickler Syndrome and RRD in Saudi Arabia

In SA, the prevalence of SS has not been documented previously. However, the percentage of RRD in patients with SS in SA is still lacking, as the available studies were conducted on patients with SS who already had RRD [27,28]. A retrospective case series involving 12 children suspected of having Stickler syndrome revealed that retinal detachment (RD) was found only in 4 of them after undergoing confirmatory genetic testing. In these 4 children with RRD, genetic testing revealed that 3 of them had one copy of a harmful change in the *COL11A1* gene, while 1 child had two copies of a harmful change in the *LRPAP1* gene [29]. RRD in SS is complex and difficult to manage with a high recurrence rate and low success rate. Combined scleral buckling and vitrectomy were found to have the best outcome; however, the outcome for the first surgery remains low [27,28]. There have been reports of prophylaxis treatment (laser photocoagulation or cryotherapy) [24,30]. However, Table 1 summarizes several phenotypes that manifest high myopia in association with RRD.

### 2.3. Knobloch Syndrome

Knobloch syndrome (KS) (OMIM: #267750, #608454) was first described by Knobloch et al. in 1971 [39]. It is a rare AR disorder that is caused by a mutation in *COL18A1* that is typically characterized by a triad of high myopia, vitreoretinal degeneration with high risk of RD, and occipital lobe defect [40,41]. Type XVIII collagen is found in Bruch’s membrane, lens capsule, basal membrane of the iris, aqueous humor, vitreous, and retina. [18] Knobloch syndrome has identified at least 20 polymorphic changes in the *COL18A1* gene, which includes 43 exons and has three distinct isoforms in humans [7,12]. In addition, *ADAMTS18* may represent a different pathogenic locus in patients with Knobloch syndrome [42]. Although the ocular and occipital lobe abnormalities are the two hallmark features of KS, a wide phenotypic spectrum of ocular and neurological abnormalities clearly exists in the literature [42,43]. Khan AO et al. [41] reported more details of the ophthalmological features in children with genetically proven KS in SA. Manifestations include cryptless irides, ectopia lentis, high myopia ranging from −10 to −20 diopters, characteristic vitreoretinal degeneration that appears as diffuse severe atrophic retinal pigment epithelium with prominent choroidal vessels, which is pathognomonic of KS, atrophic lesions in the macula with or without punched-out appearance, and fibrillary white vitreous. There have also been reports of peripheral avascular retina and macular hole-related retinal degeneration [34,44]. Brain malformations are variable, including occipital lobe abnormalities (may or may not be present, ranging from scalp defects such as cutis aplasia to bone defects to encephalocele), developmental delay, seizure, and cognitive impairment [45,46]. Additional findings that further expand the clinical spectrum of KS include lung hypoplasia, duplication of the renal collecting system, hyperextensible joints, and dysmorphic findings such as midface hypoplasia, flat nasal bridge, dental abnormalities, high-arched palate, or micrognathia [43,47,48].

### 2.4. Knobloch Syndrome and RRD in SA

RRD is a frequent complication of KS. A recent retrospective cohort study in SA included 50 patients diagnosed with KS based on classical clinical phenotype, with or without genetic testing. A total of 36 patients showed pathogenic variants of *COL18A1*. The study reported that 48% of these patients developed RD at a mean age of 6 years [49]. Macular hole-related RRD in KS is common in SA [34]. Alzaben KA et al. reported that one-third of RRD cases in KS were secondary to macular holes [48]. PPV with SB and SO tamponade provides the best surgical outcome [49].

### 2.5. Pierson Syndrome

In 1963, Pierson and associates initially described a condition of severe microcornea with fatal congenital nephrotic syndrome (CNS) in two siblings. Zenker M et al. later named this condition Pierson syndrome (PS) and discovered that it is an AR disorder caused by a change in the *LAMB2* gene [50]. The *LAMB2* gene encodes laminin beta 2, which is expressed in the glomerular basement membrane, neuromuscular junctions, and within ocular tissue, causing a wide range of clinical abnormalities in these organs [51]. Ophthalmological signs that have been described in PS include microcornea, posterior embryotoxon, megalocornea, flat iris, iris hypoplasia, hypoplastic ciliary body, shallow anterior chamber, cataract, posterior lenticonus, microphakia, RD, persistent fetal vasculature, and glaucoma [8]. Interestingly, high myopia in combination with peripheral retinal ischemia is a characteristic posterior segment feature in Pierson syndrome and Pierson syndrome needs to be considered in patients who manifest these retinal features.

### 2.6. Pierson Syndrome and RRD in SA

A thorough description of posterior segment manifestations in PS was reported by AlTaisan A et al. [8] in their retrospective study of 16 eyes with PS in SA, including high myopia with its features (tessellated fundus, pale disk with unidentified cup, abnormal emanation of the retinal vessels from the optic disk (situs inversus), and peripapillary chorioretinal atrophy), RRD, and features of abnormal retinal vascularization (peripheral avascular retina on fluorescein angiography, aberrant course of the temporal arcades, straightened nasal retinal blood vessels, and tortuous retinal blood vessels). They also reported retinal ischemia, retinal/iris neovascularization, hyphema, vitreous hemorrhage, and neovascular glaucoma [30,52]. Although the most distinctive ocular anomaly in PS is microcornea [8], Alshamrani et al. [30] reported that none of the eyes in their study had microcornea. Renal involvement is typically described as a severe form of CNS that is rapidly progressive, leading to early chronic end-stage kidney failure and even death, as it is described in the original paper by Pierson [53]. However, several studies thereafter reported milder or variable forms of the CNS or even an absent nephrotic range of proteinuria [54,55,56]. Interestingly, the severity of the renal phenotype is not always parallel to that of the ocular phenotype [56]. In SA, two retrospective studies consisting of 22 and 16 eyes, respectively, found that the incidence of RRD in children with genetically confirmed PS was 63.6% and 43.75% [9,54]. Comparing this figure to another retrospective study with a larger number of patients who were molecularly diagnosed with PS (34 eyes) in which only 3 eyes developed RD, this figure is considered high [8]. RRD mostly occurs during childhood between the ages of 6 and 7 years [30,54]. Different surgical methods were used to fix RRD in PS, like SB with cryo, PPV with SO tamponade, and a mix of SB with PPV and SO tamponade; nearly all of these methods had cases of recurrence and needed more surgery, and no research has compared which method works best.

### 2.7. Non-Syndromic Inherited High Myopia

Non-syndromic inherited high myopia is primarily inherited in an autosomal dominant manner, but it can also occur infrequently as recessive or X-linked inheritance patterns [57]. Thus far, researchers have linked three genes to this condition: cathepsin H (*CTSH*, OMIM: 116820) and low-density lipoprotein receptor-related protein-a. associated protein 1 (*LRPAP1*, OMIM: 104225), and *LEPREL1* (Prolyl 3-Hydroxylase 2, OMIM: 610341) [58,59,60,61,62].

### 2.8. Recessive LRAPAP1-Related Myopia

*LRPAP1* is a broadly expressed gene and encodes low-density lipoprotein receptor-related proteins, which serve as a chaperone for lipoprotein receptor-associated proteins (LRP1 and LRP2). Mutations in *LRPAP1* lead to the perturbation of transforming growth factor beta (TGF-β), which disrupts the scleral extracellular matrix, resulting in axial length elongation [59]. Additionally, higher levels of TGF-β can change how flexible the retina is, making it stiffer and more likely to develop tears and detach from the back of the eye. The changes caused by *LRAPAP1* mutations include a nearsighted eye appearance with serious widespread damage to the retina and a cone-shaped area around the optic nerve head. Other features like vitreoretinopathy, lacquer cracks, neovascularization, and RRD-predisposing peripheral degeneration were not reported [62].

### 2.9. LRAPAP1 and RRD in Saudi Arabia

In Saudi Arabia, Magliyah et al. [31] and his group reported an incidence of RRD in 42% of a cohort (12 patients) with non-syndromic recessive *LRAPAP1*-related myopia; all of these patients were legally blind at the last clinic follow-up, with vision ranging from 20/300 to NLP. Two cases had inoperable total retinal detachment with PVR grade C, while the remaining four cases underwent vitrectomy [32]. Retinal detachment (RD) and proliferative vitreoretinopathy (PVR) are the main reasons for vision problems in *LRAPAP1*-related myopia; it is advised to identify these issues early, monitor them closely, and use preventive laser treatment to avoid losing vision.

### 2.10. Recessive LEPREL1-Related Myopia

The *LEPREL1* gene is essential for eye development by encoding prolyl 3-hydroxylase 2 (P3H2), a 2-oxoglutarate-dependent dioxygenase that hydroxylates collagens. This enzyme is widely expressed in collagen fibril-containing tissues, such as the lens capsule of developing mouse embryos [63]. Prolyl hydroxylation is a critical post-translational modification for various types of collagens, although its precise function and the exact mechanism are not well understood [64]. Additionally, P3H3 interacts with basement membrane collagen (IV), which is predominantly expressed at the retinal internal limiting membrane (ILM), the main basement membrane in the eye; therefore, a mutation in the *LEPREL1* gene can compromise ILM integrity by disrupting the recognition and hydroxylation of collagen IV at the ILM during eye development, which may lead to an increase in the axial length of the eye and the development of pathological myopia [65,66,67]. Homozygous mutations in *LEPREL1* are associated with pathological myopia, early-onset cataracts, ectopia lentis, vitreoretinopathy, and retinal detachment [60,68,69,70]. These clinical features may be confused with the ocular phenotype of Stickler syndrome, making the diagnosis challenging in the absence of genetic testing [33]. Although *LEPREL1*-related phenotype is typically associated with non-syndromic myopia, Magliyah et al. [31] reported a possible association with nephropathy (microhematuria and proteinuria in 6 out of 10 patients (67%)). We recommend further research and a comprehensive workup to explore the validity of this systemic association [33].

### 2.11. LEPREL1 and RRD in Saudi Arabia

Giant retinal tears and early-onset RRD are significant causes of vision loss in LEPREL1-related myopia. Recently, Magliyah et al. [31] and their group reported an incidence of RRD in 50% of a cohort of 10 patients with nonsyndromic recessive LEPREL1-related myopia with a mean age of 14 years. This incidence is higher than the 30.7% previously reported by Mordechai et al. [59] in a cohort of 13 patients with a mean age of 22 years. The high rate of consanguinity among the Saudi population may account for this difference. To prevent ocular morbidity associated with LEPREL1-related myopia, molecular diagnosis and thorough and regular dilated fundus examination, along with prophylactic 360-degree treatment, are helpful. Compared to the global frequency, recessive *LEPREL1*-related myopia associated with RRD is markedly higher in patients from Saudi Arabia. This may be reflected by the founder variants effect, which also enhances disease penetrance and early onset among consanguineous cases [22,59].

### 2.12. Marfan Syndrome

More than 154 genes are associated with syndromic myopia; those genes have also been linked to non-syndromic myopia [71]. This includes Marfan syndrome, Ehlers-Danlos syndrome, SS, and others that share the genetic pathway present in a common syndrome [71,72]. Syndromic myopia patients also show eye problems such as retinal degeneration and cataracts, which are seen in people with Marfan syndrome [73,74]. Marfan syndrome (MFS, OMIM:154700) was first described by Antoine Marfan in 1896. It is an autosomal dominant connective tissue disorder caused by a mutation in the fibrillin-1 (*FBN1*), located at the long arm of chromosome 15 [75]. Fibrillin constitutes a key component of extracellular matrix glycoprotein and is widely expressed in ocular tissues such as zonules, lens capsules, ciliary body, sclera, choroid, and Bruch’s membrane [76]. Mutation of *FBN1* leads to decreased and disrupted incorporation of fibrillin into the connective tissue matrix, which explains the ocular phenotype of MFS [77]. MFS is characterized by ocular and systemic manifestations that notably involve cardiac and musculoskeletal systems. Ocular manifestations of MFS include axial myopia, bilateral ectopia lentis, early-onset cataract, iris abnormalities, vitreous liquefication, lattice degeneration, retinal breaks, retinal detachment, maculopathy, posterior staphyloma, and glaucoma [77,78,79]. Systemic manifestations include aortic dilation, valvular problems, tall stature, dolichostenomelia, generalized joint laxity, arachnodactyly, chest deformities, a high-arched palate, and scoliosis or kyphoscoliosis [80]. While cardiac complications are the most life-threatening aspect of MFS [81], retinal detachment represents the most common and serious visual complication, with an incidence range from 8% to 25% in affected individuals [82]. Bilateral retinal detachment was observed in 30–43% of the cases [83]. The pathogenesis of retinal detachment in MFS cases is multifactorial, involving a subluxated or dislocated lens that exerts traction on the vitreous base, leading to small breaks and subsequent RRD. Additionally, vitreous liquefaction, post-vitreous detachment, and abnormal vitreoretinal adhesion predispose patients to multiple breaks or giant retinal tears (GRT) [82,83,84,85]. MFS remains a clinical diagnosis when sufficient features are identified. Early diagnosis and prompt referral to both cardiologists and ophthalmologists are crucial for improving the life expectancy and overall quality of life of affected individuals. Regular follow-up and correction of refractive errors are essential to prevent amblyopia. Furthermore, we recommend meticulous fundus examination and prophylactic laser treatment for high-risk patients to prevent RRD. Advances in surgical technique and instruments have improved the surgical outcome, with retinal reattachment success rates in uncomplicated cases ranging from 75% to 89%, comparable with the general population [82,84,85,86,87].

### 2.13. Marfan Syndrome and RRD in Saudi Arabia

Ocular complications commonly associated with MFS include RRD, which has been observed in a severe form among children in Saudi Arabia and often required surgical interventions [28,82]. The incidence of RRD in MFS patients in Saudi Arabia is limited; however, its prevalence is influenced by the genetic pool and consanguineous marriages, with higher prevalence in pediatric patients. Internationally, the incidence of RRD in MFS patients ranges from 5 to 25%, with the risk of recurrence being 30–42% [88]. To prevent vision loss, patients require meticulous fundus examination, early detection, and timely surgical intervention, all of which play a crucial role in improving long-term visual outcomes [88].

## 3. Conclusions

Consanguinity, which exacerbates inherited myopia, poses a significant health challenge in Saudi Arabia. One of the severe consequences of this is retinal detachment (RD). Syndromic types such as Stickler syndrome (SS), Marfan syndrome (MRF), and Knobloch syndrome are particularly difficult to detect and treat due to their multisystem involvement. Early detection is essential for identifying and halting the progression of the disease considering our population where we have unique population-specific founder mutations. This can be accomplished through activated genetic testing for diagnosis and screening, particularly for high-risk variants in *LRPAP1*, *LEPREL1*, and *COL18A1*. Innovative technologies like artificial intelligence (AI), machine learning, and gene therapy offer promising avenues for personalized treatment and improved outcomes. A nationwide screening program that includes genetic counseling is strongly recommended to enhance early detection and mitigate the effects of myopia. To effectively tackle inherited myopia and pave the way for precision medicine and a disease-free future in Saudi Arabia, it is essential to employ novel genetics and technologies alongside public awareness campaigns.

## Figures and Tables

**Table 1:** Genetic Variants Associated with High Myopia and Rhegmatogenous Retinal Detachment (RRD).

Disease	Gene	Allele	Study Reference No.
PS, RD, EOM	*LAMB2*	c.619T>C	[9]
PS, EOM, Neovascular Glaucoma	*LAMB2*	c.4573+1G>A	[9]
Nonsyndromic High Myopia, Rhegmatogenous RD	*LRPAP1*	c.605delT	[31]
		c.863_864delTC	
		c.1672C>T	
		c.1391G>A	
		c.292delC	
		c.679G>T	
Stickler Syndrome Type IV FEVR-like RD	*COL9A1*	c.1052C>A	[20]
		c.1349A>G	
Hemorrhagic RD, High Myopia	*COL4A1*	c.2798G>C	[32]
	*COL4A2*	c.3766C>T	
		c.1585G>A	
RD, Giant Retinal Tear, Stickler-like Phenotype	*LEPREL1*	c.1391G>A	[22]
		c.679G>T	
		c.292delC	
		c.1672C>T	
RD, Gyrate Atrophy	*OAT*	c.980C>G	[33]
Macular Hole RD, Knobloch Syndrome	*COL18A1*	c.4054_4055del	[34]
		c.355del	
Foveal Hypoplasia, Aniridia	*PAX6*	c.238_241dupACTC	[35]
RD, Early-Onset Retinal Dystrophy	*RDH12*	c.184C>T	[36]
		c.152T>A	
		c.379G>T	
		c.295C>A	
		c.806_810delCCCTG	
		c.687C>G	
		c.451C>A	
		c.451C>G	
		c.523T>C	
		c.677A>G	
		c.658+1G>A	
		c.482A>G	
		c.187+60G>A	
		c.187+54A>T	
		c.188-14insT	
		c.448+24A>G	
		c.659-25T>A	
		c.695T>G	
		c.2860C>T	
RD, Best Disease	*BEST1*	c.2953G>A	[37]
RD, Marfan syndrome	*FBN1*	c.2980G>T	[38]
		c.3037G>A	
		c.3058A>G	
		c.3095G>A	
		c.3143T>C	
		c.3157T>C	
		c.3202T>C	
		c.3217G>A	
		c.3299G>T	
		c.3302A>G	
		c.3344A>G	
		c.3350G>A	
		c.3373C>T	
		c.3388delC	
		c.3410G>C	
		c.3412T>C	
		c.3463G>A	
		c.3511T>C	
		c.3656A>G	
		c.3668G>A	
		c.3725G>A	
		c.3976T>C	

PS: Pierson syndrome; RD: Retinal detachment; EOM: Early onset myopia.
